# Supplementary material for: Reshaping of bacterial molecular hydrogen metabolism contributes to the outgrowth of commensal E. coli during gut inflammation
Source: eLife. 2021 Jun 4;10:e58609. doi: 10.7554/eLife.58609 (PMC8177889; doi:10.7554/eLife.58609)
Supplement: Supplementary file 1. [file elife-58609-supp1.docx]

**Supplementary File 1.** Primers for mutagenesis, generated for this study

| **Purpose** | **Sequence** (sequences homologous to flanking regions are printed in italics) |
| --- | --- |
| Deletion of *hyaABC* in EcN | 5’-gccatctccttgcatgc*AGAACTAACTATACGATCCATTGAGTCACTCTCTATGAC*-3’ |
|  | 5’-*gttacttatttGGGCGCACATGGTCGGCA*-3’ |
|  | 5’-*gtgcgcccAAATAAGTAACAAGGAGCGTTCATG*-3’ |
|  | 5’-caaggaatggtgcatgc*TGCGTAAGCCAGTCGTCA*-3’ |
| Deletion of *hybABC* in EcN | 5’-gccatctccttgcatgc*GTGTTAGTGGTGGATGGTTC*-3’ |
|  | 5’-*tccactacTACGTCTGTTCACGGTTATTC*-3’ |
|  | 5’-*cagacgtaGTAGTGGATGCCGACGGC*-3’ |
|  | 5’-caaggaatggtgcatgc*ACCAACAGTAAAGGTCATAGTGC*-3’ |
| Deletion of *hyaABC* in MP1 | 5’-ctagaggtaccgcatgc*AGAACTAACTATACGATCCATTGAGTCACTCTCTATGACAG*-3’ |
|  | 5’-*gttacttatttGGGCGCACATGGTCGGCA*-3’ |
|  | 5’-*gtgcgcccAAATAAGTAACAAGGAGCGTTCATG*-3’ |
|  | 5’-agctcgatatcgcatgc*TGCGTAAGCCAGTCGTCAAG*-3' |
| Deletion of *hybABC* in MP1 | 5’-ctagaggtaccgcatgc*GCCGATTGTGGATCACATC*-3’ |
|  | 5’-*cacttcgtTTATTCTCCCCGTGAGTC*-3’ |
|  | 5’-*ggagaataaACGAAGTGGTTTCAGTGAAGG*-3’ |
|  | 5’-agctcgatatcgcatgc*TGCACTCAGCATCCACGG*-3’ |
| Deletion of *frdABCD* in EcN | 5’-tcttctagaggtaccgcatg*GCGGTGTAGGTTGCGAGATG*-3’ |
|  | 5’-*gctgtgggatCGCATCGCCAATGTAAATCC*-3’ |
|  | 5’-*tggcgatgcgATCCCACAGCCCCGTACTTC*-3’ |
|  | 5’-ggagagctcgatatcgcatg*GGGCCAACGAAACGTGTC*-3’ |
| Amplification of EcN *hybABC* promoter region | 5’-acggttattcGTTATTCCGTTGCGAAGAC-3’ |
|  | 5’-tggatcccccgggctgcaggATGGATTTCTTTCGTGAGAATAATTC-3’ |
| Amplification of EcN *hybABC* coding sequence | 5’-tatcgataagcttgatatcgTTACAGAACCTTCACTGAAAC-3’ |
|  | 5’-acggaataacGAATAACCGTGAACAGAC-3’ |
